# The Austin Club

**Published:** 1885-07

**Authors:** 


The Austin Club.—It is with much pride and gratification we are able to say the profession of Austin dwell together in brotherly love and friendship. We have a County Medical and Surgical Association, in a prosperous condition, meeting regularly and doing good work; and a Microscopic Association which numbers amongst its members, not only the bulk of the physicians of Austin, but several of the Professors of the University of Texas and a number of prominent laymen who take an interest in scientific matters, and in any kind of enterprise for the good of the city. These two societies have a club room which is now being fitted up for joint use, and which, when finished, will also be the editorial room of this Journal, where our exchanges will be accessible to the members of the club and to visiting brethren. They are designed to constitute the nucleus of a library. There will be a pathological collection also and a cabinet of minerals, and other articles of interest. It is proposed to receive contributions from the physicians of the State of specimens, wet or dry, such as may be in the posession of individual surgeons, and which, being isolated and unseen except by the owners, are valueless, whereas, if donated to the Medical Club at Austin, they would help to make up an attractive and valuable collection, and would be seen and appreciated. Each donor's name will be attached to his contribution, and the specimen will be duly acknowledged and recorded, with donor's name, in a book kept by the secretary. Moreover, valuable pathological and other specimens may be sent at the expense of the club.

What city the size of Austin can make such a showing of zeal and interest in the cause in which we are engaged?

